# Assessment of Medication Adherence in Diabetes Mellitus Patients at a Tertiary Care Teaching Hospital in India

**DOI:** 10.7759/cureus.78391

**Published:** 2025-02-02

**Authors:** Srushti Shah, Preksha Barot, Hitesh Patel, Apexa Shukla

**Affiliations:** 1 Medicine, GMERS (Gujarat Medical Education and Research Society) Medical College and Hospital, Dharpur-Patan, Patan, IND; 2 Pharmacology, GMERS (Gujarat Medical Education and Research Society) Medical College and Hospital, Dharpur-Patan, Patan, IND; 3 Pharmacology, GMERS (Gujarat Medical Education and Research Society) Medical College and Hospital, Himmatnagar, Himmatnagar, IND

**Keywords:** adherence, assessment, diabetes mellitus, medication, tertiary care teaching hospital

## Abstract

Introduction: Medication adherence is a crucial aspect of patient care and is vital for the effective management of chronic diseases such as diabetes mellitus (DM). Non-adherence to medication is a pervasive issue in both developed and developing countries, posing significant challenges for healthcare providers and patients, and leading to adverse treatment outcomes and overall health complications.

Methods: After receiving approval from the institutional ethics committee, this cross-sectional, prospective study was conducted over a period of three months (December 2023 to February 2024) in the medicine outpatient department of a tertiary care teaching hospital in Gujarat, India. All patients diagnosed with DM by physicians were included. A pre-designed structured questionnaire comprising 12 questions was self-administered to DM patients. Data were analyzed using appropriate statistical tests to identify factors influencing medication adherence.

Result: A total of 390 diabetes patients were included in the final analysis. Among the participants, 232 (59%) were male, with a mean age of 58.69 ± 12.38 years. The overall medication adherence was notably high, with 356 (91.28%) participants demonstrating good adherence. The multivariable logistic regression analysis revealed a significant association between medication adherence and glycemic control. The presence of any comorbidity, current alcohol consumption, or smoking increased the odds of poor adherence by 1.78 (95%CI 1.026-3.475), 1.44 (95%CI 0.51-5.19), and 1.17 (95%CI 0.61-2.24), respectively.

Conclusion: Health education and patient counseling should be integrated into follow-up visits to enhance medication adherence. The findings of this study may serve as a foundation for developing healthcare policies and guidelines aimed at improving diabetes management. Future research should focus on evaluating additional factors influencing adherence and non-adherence, such as treatment regimen complexity, insulin resistance, and lifestyle factors, to better understand the underlying discrepancies.

## Introduction

Diabetes mellitus (DM) is a chronic metabolic disorder characterized by persistent hyperglycemia resulting from insufficient or absent insulin secretion by the pancreas, or insulin resistance [1]. Globally, DM is a leading contributor to adult mortality; accounting for approximately one in nine deaths among individuals aged 20-79 years [2]. The global prevalence in 20-79-year-olds in 2021 was estimated to be 10.5% (536.6 million) people with DM, a figure projected to escalate to 12.2% (783.2 million) by 2045 [3]. In India, approximately 74.2 individuals were diagnosed with diabetes in 2021, a number expected to rise to 124.9 million by 2045 [3]. DM imposes a substantial global and national burden, particularly through its microvascular and macrovascular complications which can severely impact patients' quality of life and healthcare systems [2].

Effective management of DM hinges on multiple factors, including strict adherence to prescribed medications, which is a cornerstone of optimal glycemic control. Adherence has been defined as the extent to which a person’s behavior, including taking medication, corresponds with agreed recommendations from a health care provider [4]. DM requires lifelong lifestyle modifications, regular exercise, and pharmacological interventions. However, despite advancements in diabetes care, medication non-adherence remains a global challenge, affecting patients in both developed and developing countries [5]. Poor medication adherence can diminish the effectiveness of therapeutic interventions, resulting in inadequate glycemic control, prolonged hospitalizations, and substantial financial burdens on patients and healthcare systems [6]. Several factors contribute to medication non-adherence, including symptomatic relief, treatment costs, complex dosing regimens, medication side effects, and patients' emotional and psychological states [7,8]. Addressing these barriers is critical for improving adherence and achieving favorable therapeutic outcomes.

Poor adherence not only exacerbates the risk of long-term complications but also undermines overall quality of life. The most common challenge encountered by physicians and other healthcare providers involved in diabetes care is to make patients adequately committed to taking their medications regularly as well as adjusting their chronic illness lifestyle [9]. Understanding the specific causes of non-adherence is essential for developing personalized interventions that produce meaningful outcomes [5]. The findings of this study will be instrumental in counseling and educating patients about the potential risks of diabetic complications and the benefits of consistent medication use. Additionally, these insights can contribute to evidence-based healthcare policies and guidelines aimed at optimizing diabetes management practices. Despite the rising prevalence of DM, data on medication adherence among diabetes patients remain scarce in many regions, including our state. Evaluating adherence levels and addressing barriers are imperative to improving patient outcomes and reducing the burden of diabetes-related complications.

This study aims to assess medication adherence in diabetic patients at a tertiary care teaching hospital and to identify the demographic and medication-related factors affecting adherence and their association with glycaemic control.

## Materials and methods

This was a cross-sectional, prospective, observational study conducted over a period of three months from December 2023 to February 2024 in the medicine outpatient department (OPD) at a tertiary care teaching hospital, GMERS (Gujarat Medical Education and Research Society) Medical College and Hospital, Dharpur-Patan, Patan, Gujarat in Western India. The study was approved by the Institutional Ethics Committee, GMERS Medical College and Hospital (approval number: MCD/Patan/IEC/138/2023 dated May 9, 2023). All participants were explained about the purpose of the study.

Inclusion and exclusion criteria

Inclusion Criteria 

The study included T2DM patients diagnosed and confirmed by physicians or documented in their electronic medical records at the study site. Participants were required to be over 18 years of age, of either gender, and could be from any ethnic background. Patients receiving one or more antidiabetic medications, along with possible additional treatments for other comorbidities, were included. The antidiabetic medications considered in the study included glipizide, glimepiride, metformin, voglibose, vildagliptin, and teneligliptin.

Exclusion Criteria

T2DM patients who were not taking any antidiabetic medications or solely on any form of insulin therapy; Patients unwilling to provide written informed consent and patients with cognitive and neurological impairment, pregnant women, critical comorbid conditions such as myocardial infarction, end-stage renal disease, liver cirrhosis, and terminal illness were excluded from the study.

Sampling method and sample size

A convenient sampling method was employed to gather data from patients who visited tertiary care teaching hospitals and data was collected at a time of convenience (mostly during OPD hours). Convenience sampling was deemed appropriate for this study due to time constraints, limited resources, and the ease of accessing the target population within the hospital setting. Based on a recent cross-sectional study with an adherence rate of 34.14% in an Indian population [10], using a margin of error of 5%, power of 80%, and 95% confidence level (CI), the sample size was calculated to be 345. Accounting for a non-response rate of 10%, the final sample size of 379 was considered adequate.

Data collection

Data was collected after obtaining written informed consent from the study participants. Confidentiality of all data was maintained throughout the study. Sociodemographic details, including initials, age, gender, religion, marital status, educational level, and occupation, were recorded. Clinical data collected included duration of diabetes, current medications with their frequency, history of smoking, history of alcohol use, random blood glucose levels, and at least one glycated hemoglobin (HbA1c) measurement within the past six months. Additionally, information on any comorbid conditions was recorded. Based on the American Diabetes Association guidelines, the standard target HbA1c level for adults is <7.0%. Hence, in this study, patients were categorized into two glycemic control groups: good control (HbA1c <7%) and poor control (HbA1c ≥7%) for assessment [11-14].

Study tool

Assessment of medication adherence was done by a self-administered, predesigned, structured questionnaire consisting of 12 questions. The questionnaire was adapted from previous studies [15-18] with minor modifications in vocabulary to improve comprehension for participants. The core content of the original questions was retained. The questionnaire was validated with input from two subject matter experts and piloted on a sample of 20 patients. These patients were not included in the final analysis.

The questionnaire was simple, quick to administer, and suitable to use in outpatient settings to assess patients’ last month’s recall for their medication-taking behavior. For ease of use, the questionnaire was translated into the local language. The questionnaire was self-administered to literate patients, while in the case of illiterate patients; the principal investigator asked each question verbally and recorded their responses. First five questions were dichotomous with responses scored as "Yes" (2) or "No" (1) point. Questions 6 to 12 used a four-point Likert scale, with responses scored as never (1), sometimes (2), often (3), and always (4) points. The total possible score ranged from 12 to 38, with higher scores indicating better medication adherence. Bloom's cut‐point was used to divide the overall adherence score as a “good” if it was equal to or greater than 78% (30 points).

Incompletely completed questionnaires were interpreted in the following way: If a single question was left unanswered, it was scored as 0, and the summed scores were expressed as usual out of a maximum of 38. If two or more questions were left unanswered, the questionnaire was excluded from the study. If a response was marked between two tick boxes, the lower score of the two options was recorded.

Data analysis

Data were recorded in Microsoft Excel 2007 (Microsoft Corporation, Redmond, Washington, United States). The collected data was analyzed using GraphPad Prism 9.4.1 for Windows (Dotmatics, Boston, Massachusetts). Relevant statistical tests were applied for data analysis. For continuous variables having a normal distribution, descriptive statistics were computed and represented as a mean ± SD, and for non-parametric variables data were expressed as the median (interquartile range (IQR): Q1-Q3). A p-value less than 0.05 was considered statistically significant. Potential factors influencing medication adherence, including age, gender, marital status, educational level, occupation, number of diabetic medications, history of smoking, history of alcohol consumption, comorbid conditions, random blood sugar (RBS), and HbA1c levels, were analyzed. These factors were analyzed using univariate analysis, where categorical variables were tested using the χ2 or Fisher's exact test, and continuous variables were tested using the Mann-Whitney U test or independent t-test. Factors found to be statistically significant in the univariate analysis were included in the multiple logistic regression analysis. The relationship between factors and medication adherence was expressed by odds ratios (ORs) with 95% CIs.

## Results

Demographic information

Out of 398 initially approached participants, eight were excluded due to incomplete answers. The final analysis included 390 patients with T2DM for medication adherence assessment. As detailed in Table 1, 232 (59%) study participants were male and 158 (40.5%) were female. The mean age of study participants was 58.69±12.38 years, ranging from 18 to 95 years. A total of 334 (85.64%) participants were Hindu and 44 (11.28%) were Muslim; 355 (91%) were married. In terms of educational attainment, 239 (61.3%) participants had completed grades 1-12, while 128 (32.8%) were illiterate. Comorbid conditions were present in 184 (47.17%) participants, with hypertension being the most prevalent comorbidity, affecting 118 (64.13%). Univariate analysis revealed that none of the sociodemographic variables, except age, were significantly associated with medication adherence (p < 0.0001) (Table 1).

**Table 1:** Association of sociodemographic factors and medication adherence (N=390) Data shown as n (%) except in age, which is shown as median (IQR). ^*^Mann–Whitney U test (U); ^#^Fischer exact test, and for the rest of the variables, Chi-squared test (χ2) was applied; **p<0.05: statistically significant. IQR: interquartile range

Variable	Good, n (%)	Poor, n (%)	Total, n (%)	P value	χ2
Gender^#^	-	-	-	-	-
Male	208 (58.4)	24(70.5)	232 (59)	0.202	-
Female	148 (41.5)	10 (29.4)	158 (40.5)	-	-
Age* (years), median (IQR)	60 (52-66)	62 (56-67)	60 (53-66)	<0.0001**	-
Education level	-	-	-	-	-
Illiterate	114 (32.0)	14 (41.1)	128 (32.5)	0.220	3.025
Up to 12^th^ pass	219 (61.5)	20 (58.8)	239 (61.2)	-	-
Graduate	23 (6.4)	00	23 (5.9)	-	-
Occupation^#^	-	-	-	-	-
Employed	178 (50)	19 (55.8)	197 (50.5)	0.634	0.22
Unemployed	178 (50)	15 (44.1)	193 (49.4)	-	-
Marital status^#^	-	-	-	-	-
Married	324 (91)	30 (88.2)	354 (90.56)	0.538	-
Single/Divorced	32(8.9)	4 (11.7)	36 (9.23)	-	-
H/O Smoking^#^	-	-	-	-	-
Yes	113 (31.7)	11 (32.3)	124 (31.79)	>0.999	0.00**
No	243 (68.2)	23 (67.6)	266 (68.2)	-	-
H/O Alcohol^#^	-	-	-	-	-
Yes	55 (15.4)	4 (11.76)	59 (15.12)	0.802	-
No	301(84.5)	30 (88.2)	331(84.8)	-	-
Comorbid condition^#^	-	-	-	-	-
Yes	172 (44.1)	12 (35.2)	184 (47.17)	0.415	0.661
No	218 (55.9)	22 (64.7)	240 (61.53)		-

Level of medication adherence among study participants

**Figure 1:**
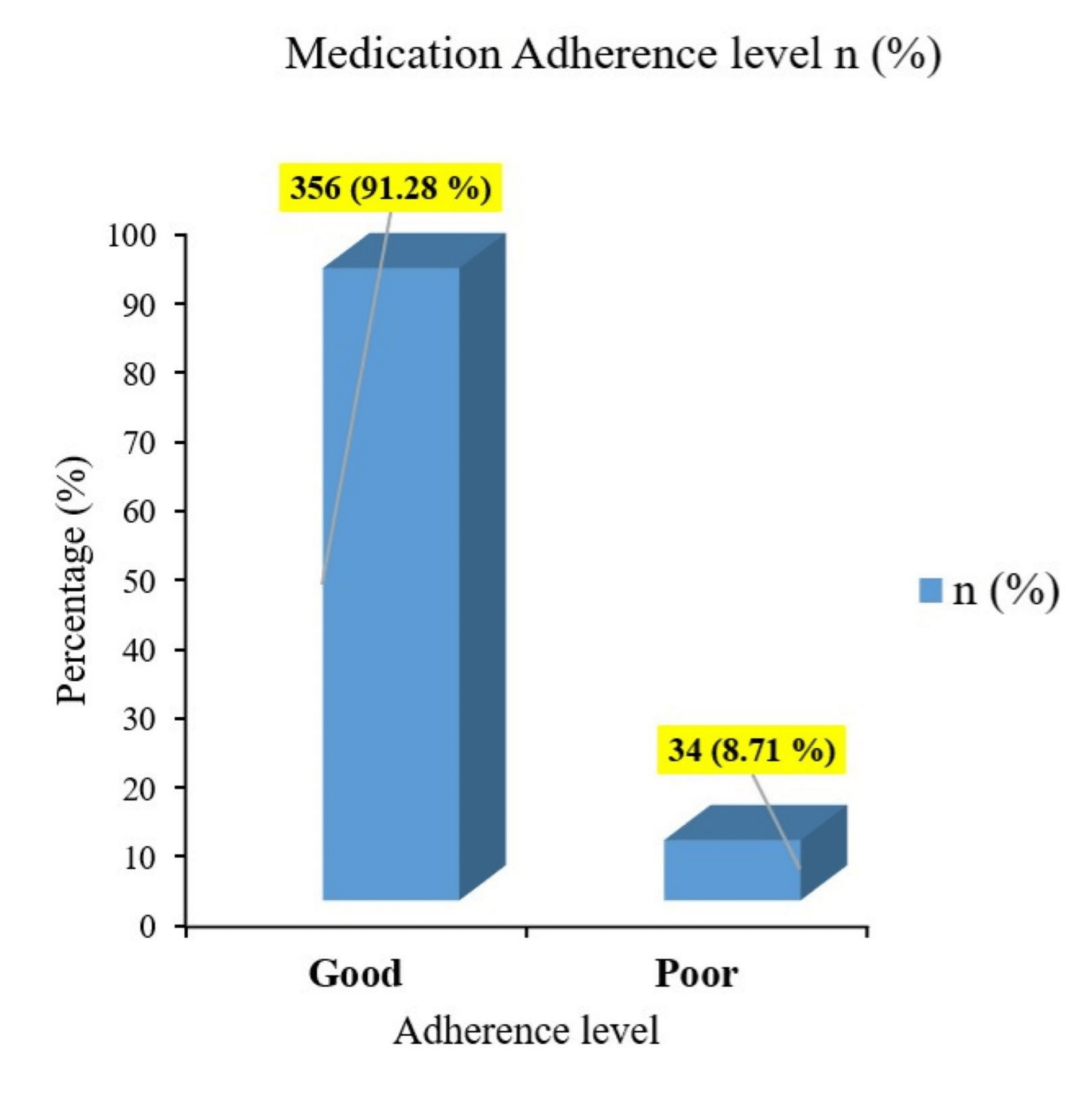
Medication adherence level in the study participants (N=390)

The responses of the study participants to individual items of the medication adherence scale are summarized in Table 2. A substantial proportion of participants selected "never" or "sometimes" for most items on the adherence scale. The study findings highlighted a notably high level of medication adherence among the participants. Specifically, out of the total sample, 356 individuals, representing 91.28% of the study population, demonstrated good adherence as illustrated in Figure 1. The average medication adherence score in the participants was 32.0 (range: 23-37) out of 38 points. Furthermore, the majority of the participants exhibited good adherence to specific behaviors, such as avoiding modification of medication dosages based on home glucose monitoring without consulting their physician and refraining from discontinuing medications due to perceiving them as unworthy of the financial expense.

**Table 2:** Medication adherence score in response to items among study participants (N=390)

Questions	Response Level	Frequency (percentage)	Mean±SD
Do you take your diabetes medications on time?	Yes	329 (84.35)	1.15±0.33
	No	61 (15.16)	
Do you obey the follow-up orders as per the advice of your physician?	Yes	341 (87.43)	1.12±0.33
	No	49 (12.56)	
Do you continue to take medications for diabetes when you are sick?	Yes	370 (94.87)	1.05±0.22
	No	20 (5.12)	
Do you carry your medications while traveling?	Yes	306 (78.46)	1.21±0.41
	No	84 (21.53)	
Do you take your medications on days when your normal routine is disturbed, For example, when you attend a family function/ festival?	Yes	259 (66.41)	1.33±0.47
	No	131 (33.59)	
Do you think medications as unworthy of money spent and discontinued?	Never	387 (99.23)	1±0
	Sometimes	3 (0.7)	
	Often	-	
	Always	-	
Do you stop/skip a dose when you are feeling better?	Never	377 (96.6)	1.04±0.23
	Sometimes	10 (2.5)	
	Often	3 (0.7)	
	Always	-	
Do you stop taking medications when you have any side effects without consulting your physician?	Never	384 (98.46)	1.02±0.21
	Sometimes	4 (1.02)	
	Often	2 (0.52)	
	Always	-	
Do you modify the dosage of medications based on home glucose monitoring without consulting your physician?	Never	387 (99.23)	1±0
	Sometimes	1 (0.25)	
	Often	2 (0.52)	
	Always	-	
Do you modify the dosage based on changes in your diet or during a fast without consulting your physician?	Never	300 (76.92)	1.35±0.71
	Sometimes	52 (13.33)	
	Often	29 (7.43)	
	Always	9 (2.3)	
Do you have difficulty following treatment plan when you have to take multiple drugs at different times of the day?	Never	332 (85.12)	1.18±0.47
	Sometimes	44 (11.28)	
	Often	14 (3.5)	
	Always	-	
Do you skip buying the medications when you have financial troubles?	Never	380 (97.43)	1.02±0.15
	Sometimes	10 (2.56)	
	Often	-	
	Always	-	

Medications used for treating participants

**Figure 2:**
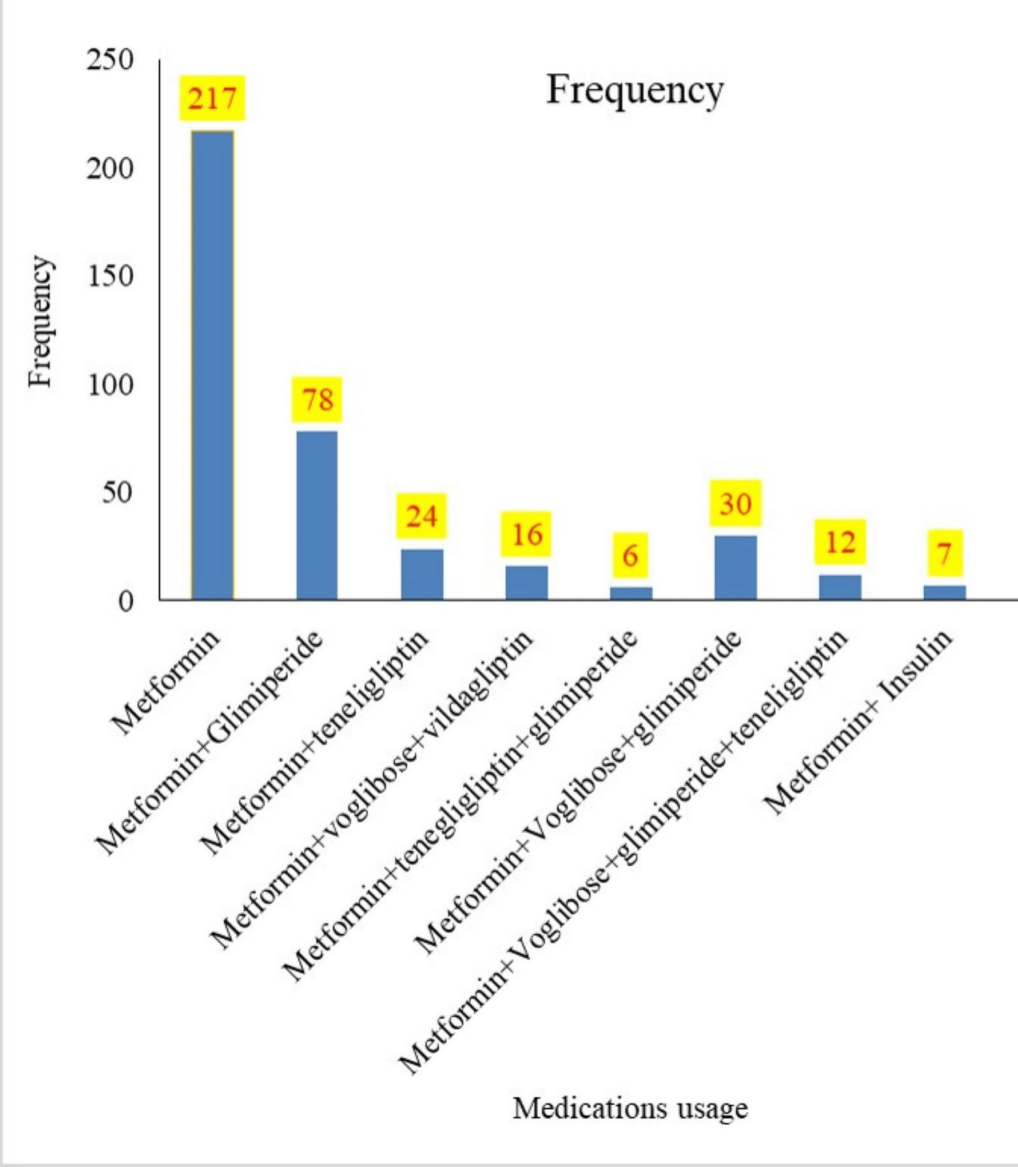
Medication used in diabetes mellitus patients

A significant proportion of participants, 217 (55.64%) were treated with metformin as monotherapy. This was followed by treatment with two oral antidiabetic drugs in 109 (27.94%) participants and three oral antidiabetic drugs in 52 (13.22%) participants, as depicted in Figure 2 and detailed in Table 3. The mean medication usage of participants who had diabetes was 1.65± 0.85 drugs with 1 median.

**Table 3:** Association of investigation and medication related factors with medication adherence in the study population (N=390) Data shown as n (%) unless specified otherwise. ^#^ Fischer exact test. **p<0.05: Statistically significant HbA1c: glycated hemoglobin

Variable	Good, n (%)	Poor, n (%)	Total, n (%)	P value	Mann–Whitney U test (U)
Duration of illness (years), median (IQR)	3 (2-5)	3 (2-4)	3 (1-5)	<0.0001**	140504
Duration of illness (years), mean ± SD	4.3 ± 3.5	3.5 ± 2.33	4.24 ± 3.43		
HbA1c^#^,mean ± SD	178± 18.3	17± 00	195 ± 18.38	0.721	-
Controlled	165 (46.3)	17 (50)	-		
Non controlled	191 (53.6)	17 (50)	-		
RBS^#^,mean ± SD	118.7± 26.69	11.33± 4.93	130 ± 31.1	0.550	-
<140 mg/dl	94 (26.4)	9 (26.4)	-		
140-199	147 (41.2)	17 (50)	-		
>200 mg/dl	115 (32.3)	8 (23.5)	-		
Drug therapy, mean ± SD	89± 79.7	8.5± 9.46	97.5 ± 89	<0.0001**	104455
One	195 (34.8)	22 (64.7)	-		
Two	101 (28.3)	8 (23.53)	-		
Three	49 (13.7)	3 (8.82)	-		
Four	11 (3.09)	1 (2.94)	-		

Glycemic control and its association with medication adherence and other variables

The average blood glucose levels among participants exceeded the target range in 123 (31.5%) participants. However, no significant difference was observed between blood glucose level and medication adherence rate. A total of 208 (53.3%) participants demonstrated poor glycemic control, as shown in Table 3. Multivariable logistic regression analysis revealed a significant association between the level of medication adherence and glycemic control. Patients with higher medication adherence were more likely to achieve better glycemic control (OR = 1.102, 95%CI: 0.73-1.65; p = 0.003).

In the final analysis, eight variables were included in the regression model using the stepwise forward likelihood method. As presented in Table 4, the model demonstrated a significant association with good medication adherence (p-value 0.007), and showed a good fit (test of goodness of fit, p-value = 0.94), with Tjur’s pseudo-R square of 0.021. The presence of any comorbidity, current alcohol consumption, or smoking increased the odds of poor adherence by 1.78 (95%CI 1.026 - 3.475), 1.44 (95%CI 0.51 - 5.19), and 1.17 (95%CI 0.61 - 2.24), respectively (Table 4). Notably, among the 262 (67.2%) participants with higher education, medication adherence was significantly improved, with an odds ratio of 1.38 (95%CI 1.01-1.927; p = 0.045).

**Table 4:** Binary logistic regression analysis for medication adherence (forward conditional LR model) The model was adjusted for gender, marital status, occupation, smoking, alcohol, HbA1C comorbid condition COR: crude odds ratio; AOR: adjusted odds ratio, LR: likelihood ratio; HbA1c: glycated hemoglobin; H/O: habit of

Variable		COR	95 % CI	AOR	95 % CI	P value
Gender	Male	0.58	(0.28 -1.2)	1.70	0.81 -3.84	0.176
	Female	1		1		
Marital Status	Married	1.35	0.48 -3.92	1.65	0.43-5.15	0.882
	Single/divorced	1		1		
Occupation	Employed	0.78	0.39-1.62	1.8	0.83- 3.95	0.135
	Unemployed	1		1		
HbA1c	Controlled	0.86	0.44-1.69	1.102	0.40-1.81	0.003*
	Uncontrolled	1		1		
H/O smoking	Yes	0.97	0.46-2.12	1.17	0.61-2.24	0.0036*
	No	1		1		
H/O alcohol	Yes	1.37	0.50-3.74	1.44	0.51-5.19	0.004*
	No	1		1		
Comorbidity	Yes	1.49	0.72-3.05	1.78	1.026- 3.475	0.0036*
	No	1		1		

## Discussion

Globally, numerous research articles and systematic reviews employing various assessment instruments have addressed the issue of poor medication adherence among diabetes patients [5,19,20]. Diabetes, a chronic metabolic disorder, requires effective management through strict diet control, consistent exercise, and regular medication to achieve optimal glycemic control and prevent future complications. Medication adherence is a critical factor influencing glycemic control and the overall health of individuals with diabetes. Noncompliance with therapeutic regimens contributes significantly to complications and increased mortality [13]. In our study, 232 (59%) participants were male, contrasting with findings from an Ethiopian study, which reported 59.3% female participants [5]. The mean age of the participants was 58.69 ± 12.38 years, with a range of 18-95 years. Similarly, an Ethiopian study reported a mean age of 52.68 ± 11.17 years, with a range of 18-82 years [5]. In terms of education, 239 (61.3%) participants in our study had completed grades 1-12, while 128 (32.8%) were illiterate. In an Ethiopian study, 21.7% were illiterate participants [5]. Additionally, 184 (47.1%) participants in our study had T2DM with comorbid conditions, of which hypertension was the most common, affecting 118 (64.13%) of these individuals. A similar finding was reported by Sahoo et al., who identified hypertension as the most common comorbidity, present in 45.6% of patients [10].

Our study participants’ medication adherence was significantly good (91.28%), with an average medication adherence score of 14 (range 23-37) out of 38 points. A study by AlQarni et al. revealed the mean score for overall adherence to anti-diabetic medications was 26.34 ± 5.6 out of 33 in Saudi Arabia patients [14]. A study by AlShayban et al revealed an average adherence score of 25.3 out of a total of 33 (median 27, IQR 7), and a third of patients (n=105, 33%) had high adherence followed by the same number of patients who were partially adherent in Saudi Arabia [21]. So the overall adherence score was lower in our study as compared to those reported in previous studies. In contrast, a study by Lee et al. in a Chinese population reported that 57.1% of participants had low medication adherence [19]. An Indian study revealed low adherence rates with only 1% showing high, while 34% moderate and 65% low medication adherence [20]. Alqarni et al. reported adherence rates of 35.7% high, 42.9% intermediate, and 21.4% low among diabetes patients [14]. A systematic review from low and middle-income countries reported medication adherence rates ranged from 26.0% to 97.0% [22]. The observed discrepancies between our findings and those of other studies may be attributed primarily to variations in operational definitions and sample sizes. Additionally, differences in sociodemographic factors, lifestyle behaviors, cultural and religious beliefs, financial status, healthcare services, diabetes management practices, and ongoing health policies are likely to contribute to the observed variability in adherence rates.

Most of the participants (99.23%) reported good adherence to specific behaviors such as avoiding modification of medication dosages based on home glucose monitoring without consulting their physician and refraining from discontinuing medications due to perceiving them as unworthy of the financial expense. Our findings suggest that patients trust their physicians in managing their condition. Since physicians provide initial care and plan follow-up visits, fostering effective communication, mutual respect, and shared decision-making can promote adherence to treatment. Consistent follow-ups and ongoing physician-patient interactions help strengthen the relationship, offering opportunities to identify and address adherence challenges and make necessary adjustments to treatment plans.

Medication adherence was positively correlated with patient age. Univariate analysis revealed that adherence improved significantly with increasing age (p<0.0001), a trend consistent with findings from studies conducted among diabetic populations globally [5,14,19,23]. This association may stem from the higher prevalence of comorbid conditions in older patients, necessitating the use of multiple medications [24]. Additionally, long-term experience with antidiabetic therapies among older patients may contribute to enhanced awareness regarding the importance of adherence. However, managing multiple comorbid conditions often poses challenges for older adults, and previous studies have identified this as a major barrier to medication adherence [21,24-25]. In the current study, binary logistic regression analysis revealed that the presence of comorbidities increased the odds of poor adherence by 1.78 times (95%CI: 1.026-3.475). Similarly, Sahoo et al. reported a 3.26-fold increase in the likelihood of poor adherence in the presence of comorbidities (95%CI: 1.93-5.50) [10], a finding supported by research conducted in Ethiopia [26]. Furthermore, a study by Venkatesan et al. indicated that individuals with hypertension were 1.6 times more likely to exhibit poor adherence (95%CI: 1.04 - 2.5) [27].

Younger patients may exhibit poorer medication adherence due to a lack of awareness about DM and its long-term complications. Additionally, younger individuals are more likely to be employed, and their working hours may interfere with adherence to prescribed regimens. A similar observation was reported in a study conducted in Singapore [19]. This finding underscores the importance of targeted educational initiatives aimed at increasing awareness among the younger population regarding self-care practices and the long-term implications of diabetes. In the present study, the duration of illness was significantly associated with poor medication adherence (p<0.001). Furthermore, an increase in the number of prescribed medications was negatively correlated with adherence. Participants with a higher number of prescribed drugs were less likely to follow their treatment regimens, with a significant association between polypharmacy and poor adherence (p<0.0001). This observation aligns with previous studies, which reported that pill burden adversely impacts treatment adherence [5,21]. The complexity of antidiabetic regimens may present additional challenges to adherence, as patients often struggle to manage intricate medication schedules. Among older adults, forgetfulness has been identified as a potential factor contributing to poor medication adherence [22]. These findings highlight the need for simplified medication regimens and adherence support strategies to improve outcomes, particularly in vulnerable populations.

In our study, 123 (31.5%) participants exhibited average blood glucose levels far higher than the target range; however, this was not statistically significantly associated with medication adherence. Poor glycemic control was observed in 208 (53.3%) participants. According to the American Diabetes Association (ADA), maintaining an optimal HbA1c level reflects effective glycemic control over the preceding two to four months [12]. Based on this criterion, we analyzed the relationship between adherence scores and HbA1c levels. Our findings revealed a negative correlation between HbA1c levels and medication adherence scores, indicating that participants who consistently adhered to their medication regimens achieved better glycemic control. The multivariable logistic regression model further confirmed this relationship, with adherence being significantly associated with lower HbA1c levels (p=0.003). These results are consistent with prior studies [14,19,21,23]. Khotkar et al. reported that only 19% of individuals with T2DM achieved optimal glycemic control, while 81% experienced uncontrolled glycemic levels [20]. Factors such as dietary modifications, regular physical activity, and appropriate pharmacological therapy play a critical role in determining a patient's glycemic control [26]. These findings underscore the need for a comprehensive approach to diabetes management to improve outcomes.

In our study, current alcohol consumption and smoking were associated with increased odds of poor medication adherence, with ORs of 1.44 (95%CI: 0.51-5.19) and 1.17 (95%CI: 0.61-2.24), respectively. Similarly, a study by Venkatesan et al. reported that current alcohol consumption raised the likelihood of poor adherence by 2.35 times (95%CI: 1.03-5.36) [27]. In contrast, Yosef et al. found that non-alcoholic participants had 2.3 times higher odds of good medication adherence [26]. These findings suggest that substance use, or association with substance-using peers, may contribute to a disorganized lifestyle that hampers adherence to prescribed regimens and impairs judgment regarding diabetes self-care activities. Educational attainment was another significant factor influencing medication adherence. Among the highly educated participants (67.9%), adherence was significantly higher, with an OR of 1.38 (95% CI: 1.01-1.927; p=0.045). This finding aligns with a study from Saudi Arabia, which observed a strong association between higher education levels and medication adherence (χ² = 46.02, p<0.01) [21]. Similarly, an Indian study also reported a significant correlation between educational attainment and adherence [27], consistent with findings from other research [28,29]. Educated individuals are more likely to understand the implications of non-adherence and the long-term complications of diabetes, thereby promoting better adherence behaviors [15]. In contrast, a study conducted in Singapore did not find a significant association between educational level and medication adherence [19], highlighting the potential influence of contextual and cultural factors on adherence behaviors. These findings emphasize the importance of personalized interventions to address diverse determinants of adherence.

In our study, 217 (55.64%) patients were treated with metformin. Metformin is widely recognized as a first-line therapy for T2DM due to its efficacy in lowering blood glucose levels. It is effective both as monotherapy and in combination with other antidiabetic agents. The widespread use of metformin can be attributed to its affordability, availability, and accessibility leading to good medication adherence among patients. Additionally, metformin has been shown to improve glycemic control while reducing the risk of hypoglycemic episodes and weight gain compared to sulfonylureas and insulin [30]. However, gastrointestinal side effects, including flatulence, nausea, and diarrhea, may hinder adherence in some patients, potentially leading to suboptimal glycemic control [22]. These findings underscore the need for patient education and monitoring to mitigate side effects and promote adherence to metformin therapy.

The present study has several limitations. The study relied on a self-administered questionnaire to assess medication adherence and patients might have over-reported adherence due to fear of judgment or under-reported due to forgetfulness. Moreover, it did not assess the outcome of medication adherence. Since adherence data relied on participants’ recall, the actual adherence rate could be lower than the reported findings. Memory-related challenges in recalling long-term medication-taking behaviors may have influenced responses. However, this effect was mitigated by limiting the recall period to the previous month. Selection bias is another potential limitation, as participants who regularly visited outpatient departments for consultations were likely to be more health-conscious than the general population. Lastly, social desirability bias may have been introduced through the self-report nature of the questionnaire, as patients may overstate their adherence to please healthcare providers. Future research with alternative methods can include electronic monitoring devices like medication event monitoring systems (MEMS), which record the time and date a medication container is accessed, offering objective data. Pharmacy refill records can provide insights into adherence patterns over time, serving as an objective measure. Biomarkers, such as drug levels in blood or urine, can assess adherence, though they may be invasive and costly. Additionally, digital health technologies, including mobile health apps, or wearable devices, can enable real-time adherence monitoring and send reminders to patients.

## Conclusions

In our study, the overall medication adherence among T2DM patients was notably high (91.28%). Factors such as age, chronic illness, and pill burden played a significant role in determining adherence rate. Younger age, longer duration of illness, and a higher number of prescribed medications were significantly associated with poor adherence. Additionally, poor adherence was significantly associated with elevated HbA1c levels, the presence of co-morbid conditions, and lifestyle factors such as alcohol consumption and smoking. These results highlight the need for integrating health education and patient counseling into routine follow-up visits to improve adherence and optimize diabetes management. The findings of this study can serve as baseline data for policymakers, government organizations, and other stakeholders involved in evaluating current medication use policies. Based on these findings, we recommend that health institutions establish dedicated DM clinics to improve follow-up care for chronic patients.

To better understand patient adherence and address the various challenges related to adherence and non-adherence, further evaluation is needed on factors such as treatment regimen complexity, insulin resistance, and lifestyle. Additionally, this study did not assess the impact of the physician-patient relationship on medication adherence, although a positive relationship is known to enhance adherence and patient satisfaction. Therefore, larger follow-up studies are needed to explore the role of physicians' empathy in improving adherence among diabetic patients. Qualitative research, including in-depth interviews, could also provide valuable insights into patients' beliefs, attitudes, and experiences regarding medication adherence, uncovering unique barriers and facilitators.
